# Cold Stress Tolerance in Psychrotolerant Soil Bacteria and Their Conferred Chilling Resistance in Tomato (*Solanum lycopersicum* Mill.) under Low Temperatures

**DOI:** 10.1371/journal.pone.0161592

**Published:** 2016-08-31

**Authors:** Parthiban Subramanian, Kiyoon Kim, Ramasamy Krishnamoorthy, Anbazhagan Mageswari, Gopal Selvakumar, Tongmin Sa

**Affiliations:** 1 Department of Environmental and Biological Chemistry, Chungbuk National University, Cheongju, Chungbuk 361–763, Republic of Korea; 2 School of Biosciences and Technology, VIT University, Vellore 632014, Tamil Nadu, India; Universite Paris-Sud, FRANCE

## Abstract

The present work aimed to study the culturable diversity of psychrotolerant bacteria persistent in soil under overwintering conditions, evaluate their ability to sustain plant growth and alleviate chilling stress in tomato. Psychrotolerant bacteria were isolated from agricultural field soil samples colleced during winter and then used to study chilling stress alleviation in tomato plants (*Solanum lycopersicum* cv Mill). Selective isolation after enrichment at 5°C yielded 40 bacterial isolates. Phylogenetic studies indicated their distribution in genera *Arthrobacter*, *Flavimonas*, *Flavobacterium*, *Massilia*, *Pedobacter* and *Pseudomonas*. Strains OS211, OB146, OB155 and OS261 consistently improved germination and plant growth when a chilling stress of 15°C was imposed and therefore were selected for pot experiments. Tomato plants treated with the selected four isolates exhibited significant tolerance to chilling as observed through reduction in membrane damage and activation of antioxidant enzymes along with proline synthesis in the leaves when exposed to chilling temperature conditions (15°C). Psychrotolerant physiology of the isolated bacteria combined with their ability to improve germination, plant growth and induce antioxidant capacity in tomato plants can be employed to protect plants against chilling stress.

## Introduction

Seasonal snow and soil frost covers 55% of the northern hemisphere and freeze-thaw cycles in soils of these regions are expected to increase in severity and frequency along the next century [1]. Such natural elements which cause intermittent or seasonal drop in soil temperatures, significantly affect plant growth and other soil-biotic activities [2]. The term chilling temperatures pertain to low but non-freezing (0–15°C) temperatures that are common during early spring season in temperate regions which often substantially compromise plant productivity [3,4]. Further, such conditions also lead to crop loss by encouraging growth of saprophytic fungi in addition to disturbing the natural soil nutrient cycling thereby reducing soil fertility [1,2]. Exposure to low temperatures disrupts the cellular homeostasis in plants and reactive oxygen species (ROS) are some of the major products of stress-induced cellular changes [5]. ROS such as hydrogen peroxide (H_2_O_2_), singlet oxygen (O_2_^-^) and HO^.^, damage biomacromolecules including proteins, lipids, carbohydrates and DNA, ultimately leading to total cell death in plants [6,7].

Tomato (*Solanum lycopersicum*) is a subtropical crop grown under greenhouse conditions in temperate regions and is physiologically sensitive to chilling stress at all its stages of growth. Most of the commercially grown cultivars of tomato are sensitive to temperatures below 15°C and a drop below 12°C usually inhibits its growth [8,9]. In tomato, low chilling temperatures of less than 18°C may affect growth, truss formation, anthesis and fruit ripening [9]. General symptoms that occur in response to chilling injury in horticultural plants including tomato are surface lesions, discoloration due to loss of chlorophyll, plant death and accelerated senescence [10]. ROS are often found to cause the above mentioned symptoms and therefore it can be understood that chilling injury caused in tomato plant is an outcome of its ROS accumulation. There exist several reports in the recent past where psychrotolerant bacteria improved plant growth under chilling [11–16]. However, the effect of such plant growth promoting bacteria (PGPB) in regulating specific plant responses that are accountable for chilling tolerance have not been studied widely [17]. In the present work, an attempt was made to isolate psychrotolerant bacteria from soils collected at agricultural fields during winter, examine their physiological adaptations to cold and further employ them for chilling stress alleviation in tomato plants by improving the plant endogenous ROS scavenging system.

## Materials and Methods

### Soil sampling and physiochemical properties

Soil samples were collected from experimental fields of Chungbuk Agricultural Research and Extension Services, Ochang-eup, South Korea (36°43' N; 127°27'E) during late winter (January, 2012). Sample collection sites were fallow agricultural fields with an average soil temperature of 0.1 to 2°C and the mean maximum air temperature of Korea for the month of January was 3.8°C [18]. Soil from a depth of 10 up to 15 cm were collected in triplicates and stored at 5°C until isolation of psychrotolerant bacteria. Organic red pepper, soybean and barley were cultivated in the spring before soil sample collection. The physicochemical properties of the collected soil samples are tabulated in Table 1. The soil samples were around neutral pH and non-saline in nature, and low soil temperature was considered to be the sole stress factor in the samples.

**Table 1:** Physiochemical properties of the collected soil samples.

Sampling Site	pH	EC	Organic matter	N	P_2_O_5_	K	Ca	Mg	Na	Fe
						(extractable)				
		(dS m^-1^)	(%)		(mg kg^-1^)	cmol^+^ kg^-1^				
Red Pepper Field	7.0 ± 0.02	0.8 ± 0.04	1.06 ± 0.03	0.05 ± 0.0	844.4 ± 78.6	0.2 ± 0.02	7.2 ± 0.24	0.8 ± 0.03	0.3 ± 0.02	7.9 ± 0.07
Soybean Field	7.4 ± 0.02	0.2 ± 0.01	1.5 ± 0.03	0.08 ± 0.0	881.8 ± 71.5	0.2 ± 0.01	8.3 ± 0.32	1.6 ± 0.11	0.2 ± 0.01	8.5 ± 0.16
Barley Field	7.3 ± 0.01	0.2 ± 0.01	0.76 ± 0.07	0.05 ± 0.0	540.2 ± 38.2	0.2 ± 0.01	4.3 ± 0.08	0.4 ± 0.02	0.2 ± 0.01	7.7 ± 0.09

EC electrical conductivity; N nitrogen; P_2_O_5_ soluble phosphate; K potassium; Ca calcium; Mg magnesium; Na sodium; Fe iron. Values represented are mean ± standard error of at least 3 replications experiment-^1^

### Enrichment and isolation of psychrotolerant bacteria

A modified minimal (MM) media consisting of 0.05% K_2_HPO_4_ (w/v), 0.02% MgSO_4_.7H_2_O (w/v) and 0.1% glucose (w/v) and 0.2%, (v/v), trace metal solution; pH 7.0 [19] was used for isolation of psychrotolerant bacteria. After autoclaving, filter sterilized 10% (w/v) peptone was added at 5 mL per liter when the media cooled down to room temperature (~ 20 ˚C). The trace metal mixture contained (w/v) 0.5% H_3_BO_3_, 0.04% CuSO_4_.5H_2_O, 0.2% FeCl_3_.6H_2_O, 0.4% MnCl_2_.4H_2_O, 0.2% NH_4_MoO_4_ and 0.4% ZnSO_4_.7H_2_O. For isolation, 10 g of soil sample was weighed and quickly added to 100 mL of pre-cooled sterile MM broth and allowed for enrichment of psychrotolerant bacteria by incubating at 5°C for 48 h. At the end of enrichment period, 1 mL of the broth was taken, serially diluted until 10^−7^ and 100 μL aliquots from 10^−6^ and 10^−7^ were plated on pre-cooled MM media plates and incubated at 5°C [20]. Morphologically distinct colonies, on appearance, were picked and purified by sub-culturing on the same media.

### Phylogenetic characterization

Genomic DNA was isolated and the 16S rRNA gene was amplified by PCR using universal primers 27F and 1492R. After sequencing, the isolates were identified using the EzTaxon server (http://eztaxon-e.ezbiocloud.net) on the basis of their 16S rRNA sequence data [21]. Phylogenetic analyses were performed using MEGA version 5.03 [22] after generating multiple alignments of the sequences using ClustalW. Substitutions were carried out according to the Jukes and Cantor model and clustering was performed using the neighbor-joining method. The statistical confidence of the nodes was estimated by bootstrapping using 1000 replications. Sequences of the 16S rRNA gene were deposited in GenBank and the accession numbers of the entries were obtained KF424276 to KF424315.

### Screening for psychrotolerant and plant growth promotional characteristics

To study the growth temperature relations, the isolates were initially grown in MM media broth for 72 h at 5°C and 20 μL of the cultures were spotted on agar plates followed by incubation at 5°C, 10°C, 15°C, 20°C, 25°C, 30°C, 35°C and 40°C [23]. The plates were observed for colony appearance after one week of incubation. To check the relative rate of growth, the strains were initially grown in MM media broth and 20 μL spots made on agar plates of the same media. One set of plates were incubated at 5°C and another set of plates at 25°C. The plates were observed every 24 h, and days after which visible colonies appeared were noted. To study membrane fatty acids, whole cell fatty acids of the isolates were extracted as methyl esters and analyzed using gas chromatography (GC). The fatty acids profiles of the isolates were studied for the presence of i) short chain fatty acids; ii) unsaturated fatty acids; iii) cyclic fatty acids and iv) absence of longer chain fatty acids

Expressions of plant growth promotional characteristics by the isolates were studied at 5°C. Production of indole acetic acid (IAA) by the isolates was quantified in the presence and absence of tryptophan [24]. Filter sterilized tryptophan was supplied in the medium at a concentration of 500 μgmL^-1^. The ability to solubilize insoluble phosphate was carried out in NBRIP-BPB plates [25]. Siderophore production was studied on CAS agar plates prepared according to Alexander and Zuberer [26]. ACC deaminase activity was determined by growing the bacterial isolates on nitrogen-free medium amended with 3 mM ACC as nitrogen source [27] and the amount of α-ketobutyrate produced by the enzymatic hydrolysis of ACC was estimated. Salicylic acid production in culture supernatants was determined as previously described [28].

### Testing tomato seed germination and seedling growth under cold conditions

Tomato seeds, *Solanum lycopersicum* cv Mill, were surface sterilized with 2% sodium hypochlorite containing 0.02% Tween20 for 5 min, 70% ethanol for 1 min followed by rinsing thrice with sterile deionized water, 3 min for every wash. Aliquots (100 μL) of water from the final wash were spread on nutrient agar to ensure efficiency of sterilization. Seed treatments consisted of soaking surface sterilized seeds in sterile media or late log phase cultures of the isolates for 4 h. At the end of seed treatments, 10 seeds were transferred to each petriplate containing sterile moist filter papers and treatments were maintained in triplicates. The plates were then moved to a plant growth chamber maintained at 15°C under dark conditions. At the end of 10 days, the germination percentage was calculated.

In another set of experiments, bacteria-treated and non-treated tomato seeds were germinated at 25°C/25°C (day/night) conditions in a controlled plant growth chamber and the temperature reduced to 15°C / 15°C on the 6^th^ day when all the seeds were found to have germinated. The low temperature exposure was imposed over a period of one week and the seedlings were observed for shoot and root growth. From the results of these experiments, strains *Pseudomonas frederiksbergensis* OS211, *Flavobacterium glaciei* OB146, *P*. *vancouverensis* OB155, and *P*. *frederiksbergensis* OS261 were selected based on their ability to improve seed germination and early growth under chilling stress (S1–S3 Figs).

### Bacterial priming and assessment of chilling resistance in tomato plants

Tomato seeds were surface-sterilized and treated with bacterial cultures *Pseudomonas frederiksbergensis* OS211, *Flavobacterium glaciei* OB146, *P*. *vancouverensis* OB155, and *P*. *frederiksbergensis* OS261 as described above. Germination was carried out in seedling trays maintained at 25°C/25°C (day/night) conditions. One week old seedlings were transferred to pots in greenhouse and continued to be maintained under same temperature conditions for two more weeks and at the end of 21 days the pots were exposed to chilling temperatures of 15°C/ 15°C by transferring to a temperature controlled plant growth chamber. The plants were harvested after one week of chilling treatment.

### Membrane permeability–electrolyte leakage and malondialdehyde content

Membrane permeability was studied in terms of electrolyte leakage and malondialdehyde content in the leaves. To study electrolyte leakage, six fully grown leaves were rinsed, blotted dry and placed in conical tubes containing 15 mL of double distilled water. The tubes were incubated for 24 h at 25°C. After incubation, conductivity (E1) was measured with a conductivity meter. Subsequently, the tissue was placed in a 100°C water bath for 30 min, and then cooled to 25°C. A second conductivity measurement was made (E2). The electrical conductivity of deionized water was also measured (E0). The relative electrolyte leakage (REL) was calculated as follows [14]

$$Relative\;electrolyte\;leakage\;(\%)=\frac{E1-E0}{E2-E0}\times 100$$

Malondialdehyde content was measured using protocols of Taulavuori and co-workers [29]. Leaf tissues of 0.4 g were homogenized in liquid nitrogen with a mortar and pestle and the homogenized tissue powder suspended in 6 mL of 0.1% trichloroacetic acid (TCA). The mixture was centrifuged at 10000 *×g* for 5 min and the supernatant divided in 2 tubes with 1 mL aliquots in each tube. The first tube received 4 mL of 20% (w/v) TCA and the second tube received 4 mL of 20% (w/v) TCA containing 0.5% thiobarbituric acid (TBA). The solution mixtures were heated to 95°C for 30 min and then quickly cooled in an ice bath. Centrifugation was carried out at 10000 *X* g for 10 min after cooling and the absorbance of supernatants are read at 440 nm, 532 nm and 600 nm. Malondialdehyde content was measured using its extinction co-efficient 155 mM^-1^ cm^-1^.

### ROS scavenging activity

Proline content in leaves was estimated according to protocols of Mishra and co-workers [14] with minor modifications. Leaf tissues (0.5 g) were homogenized in 5 mL of 3% sulfosalicylic acid and the homogenate centrifuged at 9000 *×g* for 10 min. The reaction mixture consisted of 2 mL of the supernatant, 2 mL of acid ninhydrin and 2mL of glacial acetic acid. The mixture was incubated in a water bath maintained at 100°C for 1 h and the reaction stopped by cooling in an ice bath. Four milliliters of toluene was used to extract the colored component present in the aqueous solution and the absorbance of the extract was measured at 520 nm. Proline content was calculated using a standard curve made using known concentrations of proline.

To measure antioxidant enzyme activity, fresh leaf samples (~500 mg) were ground to powder in liquid nitrogen using a mortar and pestle and stored at -80°C. Powdered samples (0.5 g) were homogenized on ice in 10 mL of solution containing 50 mM of potassium phosphate buffer and 1% (w/v) polyvinylpyrrolidone (pH 7.8) and kept at 4°C for 10 min. The homogenate was filtered followed by centrifugation at 4,000 *×g* for 15 min at 4°C. The supernatant was considered as the enzyme extract and stored at 4°C. Activities of superoxide dismutase (SOD), ascorbate peroxidase (APX) and glutathione synthase (GSH) in the enzyme extract were determined spectrophotometrically. SOD activity was estimated in terms of reduction in absorbance due to inhibition of the nitro-blue tetrazolium (NBT) photochemical reduction reaction by the enzyme [30]. APX activity was determined by measuring the decrease in absorbance occurring due to oxidation of ascorbic acid to dehydroascorbate [31]. APX activity was calculated using extinction co-efficient of 2.8 mM^-1^ cm^-1^ at 290 nm. GSH activity was taken as a measure of oxidation of NADPH and GSH was calculated using extinction co-efficient of 6.224 mM^-1^ cm^-1^ at 340 nm [30].

### Statistical analysis

Randomized block design was used for the seed germination, early growth and greenhouse experiments. Data from the results were normalized, subjected to analysis of variance (ANOVA) and mean significant difference were compared by t-Test (LSD) at *P*≤0.05 using SAS package 9.1.3 service pack 4. Heat map for membrane fatty acids was constructed from percentage data of the fatty acids using MS Excel.

## Results

### Isolation and phylogeny of psychrotolerant bacteria

**Fig 1:**
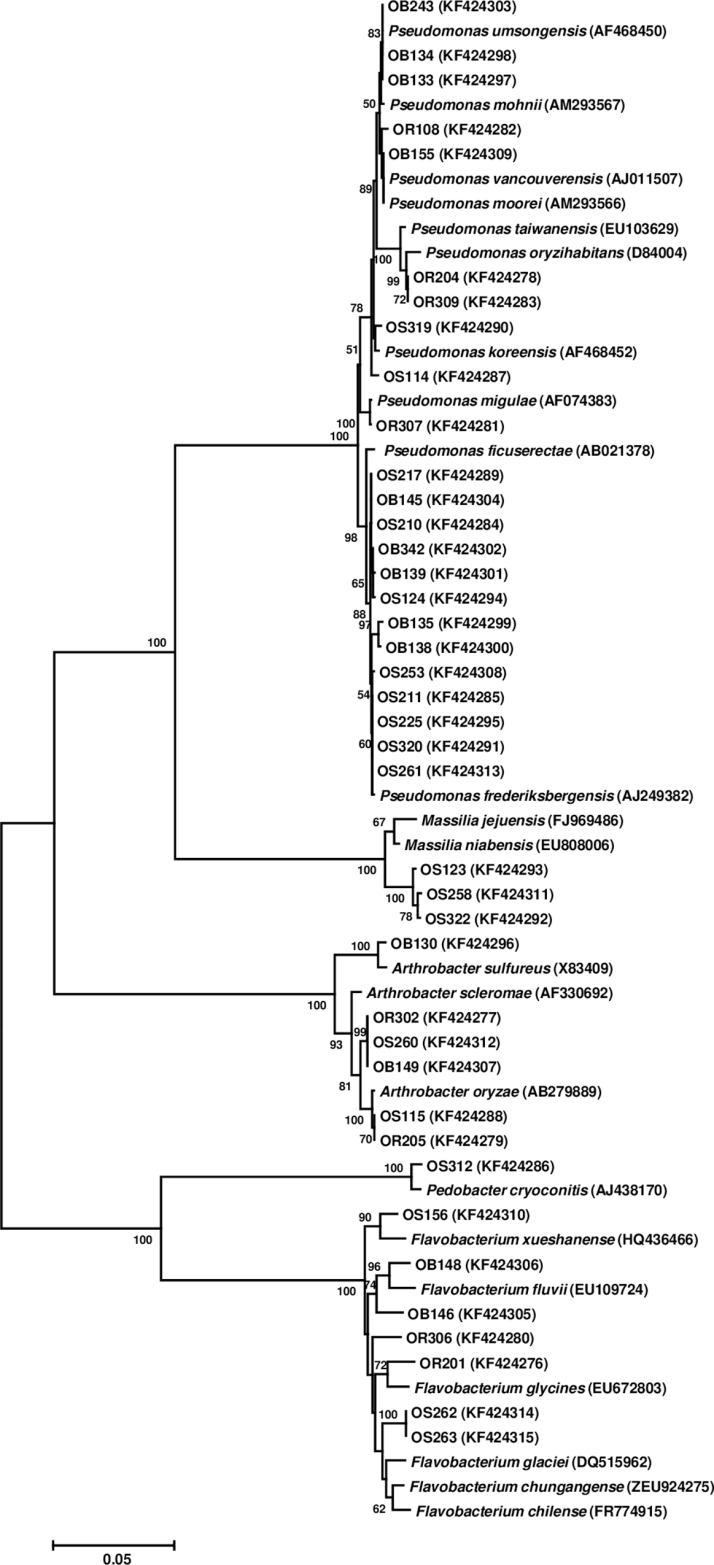
Phylogenetic diversity among the psychrotolerant bacterial isolates. Neighbor-joining tree was constructed based on 16S rRNA gene sequences. GenBank accession numbers of individual member strains are given in parentheses. Scale bar corresponds to 0.05 units of the number of base substitutions per site.

On selective isolation with MM media following an initial enrichment, visible growth was obtained on agar plates within 7–28 days. Forty phenotypically distinctive colonies were picked, purified and stored at -80°C. Phylogenetic analysis through 16S rRNA gene sequencing showed that the isolates formed distinct clades under genera *Arthrobacter*, *Flavobacterium*, *Flavimonas*, *Massilia*, *Pedobacter*, and *Pseudomonas*. Genus *Pseudomonas* presented the highest number of candidates (22) followed by *Flavobacterium* (7), *Arthrobacter* (5), *Massilia* (3) and one representative each in genera *Flavimonas* and *Pedobacter* respectively. The sequences of 16S rRNA genes were submitted to GenBank and accession numbers obtained (Fig 1).

### Psychrotolerant and plant growth promotional characteristics

**Fig 2:**
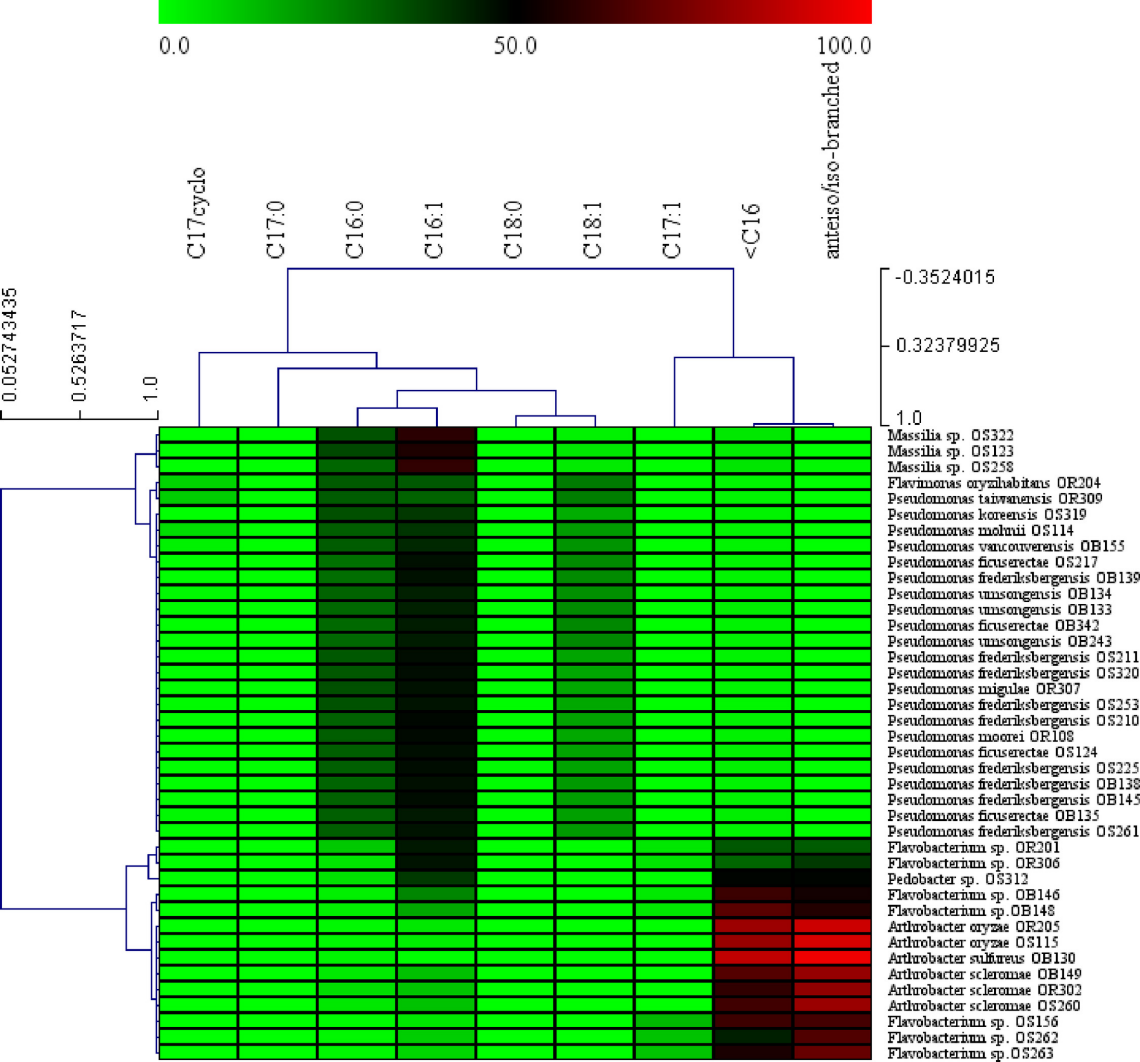
Heat map indicating the distribution of cellular fatty acids taken as markers for cold adaptation. Pair-wise Pearson correlation coefficients were used to generate heat map using MultiExperiment Viewer (MeV) software v 4.9.0. Color scale is representative of the percentage of fatty acids.

The growth temperature range of thirty-two isolates representing genus *Arthrobacter*, *Flavobacterium*, *Flavimonas*, *Pedobacter* and *Pseudomonas* ranged from 5–35°C, followed by 2 isolates of genus *Flavobacterium* exhibiting a range of 5–30°C. Six isolates representative of genus *Arthrobacter*, *Flavobacterium* and *Massilia* did not grow at temperatures beyond 25°C. Comparing the appearance of colonies on media plates, the days taken for colony appearance at 5°C was relatively longer than at 25°C (Table 2). However, most of the strains, except for those belonging to genus *Arthrobacter* and *Massilia*, showed colony appearance on the third day of incubation (Table 2). Fatty acid analysis, to study constitutive expression of fatty acid desaturases, showed C16:1 unsaturated fatty acid to be ubiquitous to all the isolates and contributed the highest percentage of cellular fatty acids. Ratio of saturated C_16:0_ fatty acids were comparatively lower than unsaturated fatty acids in all bacteria. Presence of branched iso and anteiso form fatty acids and unsaturated C_18:1_ as well as C_17:1_ fatty acids were also observed. Few of the isolates also possessed cyclic C_17:cyclo_ fatty acids which are produced by bacteria under stressful conditions. There was also a significant percentage of short chain fatty acids (<C_16_) (Fig 2).

**Table 2:** Psychrotolerant characteristics of the isolates as observed by days taken for colony appearance at 5 and 25°C and maximum temperatures of growth.

Strain	Time for colony appearance (days)		Maximum growth temperature (°C)
* *	5°C	25°C	
*Flavobacterium sp*. OR201	10	5	35
*Arthrobacter scleromae* OR302	11	4	35
*Flavimonas oryzihabitans* OR204	3	1	35
*Arthrobacter oryzae* OR205	5	1	35
*Flavobacterium sp*. OR306	5	3	35
*Pseudomonas migulae* OR307	3	1	35
*Pseudomonas moorei* OR108	2	1	35
*Pseudomonas taiwanensis* OR309	5	1	35
*Pseudomonas frederiksbergensis* OS210	3	1	35
*Pseudomonas frederiksbergensis* OS211	3	1	35
*Pedobacter sp*. OS312	6	1	35
*Pseudomonas mohnii* OS114	2	1	35
*Arthrobacter oryzae* OS115	5	1	35
*Pseudomonas ficuserectae* OS217	2	1	35
*Pseudomonas koreensis* OS319	2	1	35
*Pseudomonas frederiksbergensis* OS320	2	1	35
*Massilia sp*. OS322	9	1	25
*Massilia sp*. OS123	5	1	25
*Pseudomonas ficuserectae* OS124	2	1	35
*Pseudomonas frederiksbergensis* OS225	2	1	35
*Arthrobacter sulfureus* OB130	5	1	25
*Pseudomonas umsongensis* OB133	3	1	35
*Pseudomonas umsongensis* OB134	3	1	35
*Pseudomonas ficuserectae* OB135	2	1	35
*Pseudomonas frederiksbergensis* OB138	2	1	35
*Pseudomonas frederiksbergensis* OB139	3	1	35
*Pseudomonas ficuserectae* OB342	9	3	25
*Pseudomonas umsongensis* OB243	3	1	35
*Pseudomonas frederiksbergensis* OB145	3	1	35
*Flavobacterium sp*. OB146	5	1	30
*Flavobacterium sp*.OB148	11	5	25
*Arthrobacter scleromae* OB149	10	4	35
*Pseudomonas frederiksbergensis* OS253	3	1	35
*Pseudomonas vancouverensis* OB155	4	1	35
*Flavobacterium sp*. OS156	3	1	30
*Massilia sp*. OS258	10	1	25
*Arthrobacter scleromae* OS260	10	1	35
*Pseudomonas frederiksbergensis* OS261	3	1	35
*Flavobacterium sp*. OS262	4	1	35
*Flavobacterium sp*.OS263	5	1	35

Majority of the strains (35 strains) were Gram negative and the isolates were able to utilize sugars and sugar alcohols as sole sources of carbon. Succinate was widely hydrolyzed, but not acetate (S4 Fig). Almost all the isolated genera *Arthrobacter*, *Flavimonas*, *Flavobacterium*, *Massilia* and *Pseudomonas* except *Pedobacter* were found to utilize malate. Urease production was not observed except in the case of *Arthrobacter sulfureus* OB130 which was found to hydrolyze urea. (S1 Table). Studying the plant growth promotional characteristics at 5°C showed the effect of temperature stress on bacterial expression of such biogenic molecules. Several of the isolates were able to produce IAA, ACC deaminase, salicylic acid, siderophores and solubilize tricalcium phosphate under temperature conditions as low as 5°C. Quantitative results of plant growth promotional characteristics such as IAA production, ACC deaminase production, salicylic acid production and siderophore production are given in Table 3.

**Table 3:** Plant growth promoting characteristics of the isolates analyzed at 5°C.

Strain	IAA production (μg/mL)		ACCD activity nmol α-KB mg^-1^ protein h^-1^	Salicylic acid (mg/L)	P solubization	Siderophore production
	With trp	Without trp				
*Flavobacterium sp*. OR201	0.5 ± 0.04	0.2 ± 0	ND	1.1 ± 0.1	**-**	**-**
*Arthrobacterscleromae*OR302	1.1 ± 0.03	0.8 ± 0.09	ND	ND	**-**	**-**
*Flavimonasoryzihabitans*OR204	1.6 ± 0.08	0.8 ± 0.01	ND	1.6 ± 0.5	**+**	**+**
*Arthrobacteroryzae*OR205	0.8 ± 0.03	0.4 ± 0.02	ND	ND	**-**	**-**
*Flavobacterium sp*. OR306	ND	ND	ND	ND	**-**	**-**
*Pseudomonas migulae*OR307	1.1 ± 0.08	0.3 ± 0.03	ND	ND	**+**	**+**
*Pseudomonas moorei*OR108	1.0 ± 0.06	0.3 ± 0.03	ND	4.1 ± 0.3	**+**	**+**
*Pseudomonas taiwanensis*OR309	2.7 ± 0.19	1.5 ± 0.05	ND	ND	**+**	**+**
*Pseudomonas frederiksbergensis* OS210	0.3 ± 0.02	0.2 ± 0.02	ND	ND	**-**	**+**
*Pseudomonas frederiksbergensis* OS211	ND	ND	ND	0.5 ± 0.1	**+**	**-**
*Pedobacter sp*. OS312	ND	ND	ND	ND	**-**	**-**
*Pseudomonas mohnii*OS114	ND	ND	ND	ND	**+**	**+**
*Arthrobacteroryzae*OS115	6.2 ± 0.55	0.9 ± 0.15	ND	4.7 ± 0.5	**-**	**-**
*Pseudomonas ficuserectae*OS217	0.7 ± 0.09	0.4 ± 0.05	ND	0.3 ± 0.1	**-**	**+**
*Pseudomonas koreensis*OS319	3.2 ± 0.26	1.9 ± 0.03	0.20 ± 0.08	0.7 ± 0.1	**-**	**+**
*Pseudomonas frederiksbergensis* OS320	ND	ND	ND	0.3 ± 0.1	**+**	**-**
*Massilia sp*. OS322	ND	ND	ND	ND	**-**	**-**
*Massilia sp*. OS123	0.4 ± 0.07	0.3 ± 0.05	ND	ND	**-**	**-**
*Pseudomonas ficuserectae*OS124	ND	ND	ND	ND	**+**	**+**
*Pseudomonas frederiksbergensis* OS225	ND	ND	ND	0.3 ± 0.1	**+**	**+**
*Arthrobactersulfureus*OB130	2.7 ± 0.17	1.9 ± 0.09	ND	ND	**-**	**-**
*Pseudomonas umsongensis*OB133	1.3 ± 0.02	0.6 ± 0.1	ND	ND	**+**	**+**
*Pseudomonas umsongensis*OB134	ND	ND	ND	1.0 ± 0.1	**+**	**+**
*Pseudomonas ficuserectae*OB135	ND	ND	ND	1.4 ± 0.1	**+**	**-**
*Pseudomonas frederiksbergensis* OB138	0.5 ± 0.01	0.4 ± 0.0	4.18 ± 1.20	ND	**+**	**-**
*Pseudomonas frederiksbergensis* OB139	1.7 ± 0.01	1.1 ± 0.04	0.90 ± 0.39	ND	**+**	**-**
*Pseudomonas ficuserectae*OB342	ND	ND	ND	ND	**-**	**-**
*Pseudomonas umsongensis*OB243	2.9 ± 0.02	1.0 ± 0.03	1.73 ± 0.09	0.5 ± 0.1	**+**	**+**
*Pseudomonas frederiksbergensis* OB145	6.1 ± 0.10	4.0 ± 0.05	1.56 ± 0.17	0.8 ± 0.1	**+**	**-**
*Flavobacterium sp*. OB146	ND	ND	ND	7.3 ± 0.2	**-**	**-**
*Flavobacterium sp*.OB148	ND	ND	ND	ND	**-**	**-**
*Arthrobacterscleromae*OB149	6.1 ± 0.08	1.8 ± 0.11	4.67 ± 0.06	ND	**-**	**-**
*Pseudomonas frederiksbergensis* OS253	5.5 ± 0.09	2.4 ± 0.04	ND	ND	**+**	**+**
*Pseudomonas vancouverensis*OB155	8.0 ± 0.05	3.1 ± 0.21	32.40 ± 0.55	1.0 ± 0.1	**+**	**+**
*Flavobacterium sp*. OS156	0.2 ± 0.02	0.1 ± 0.03	ND	ND	**-**	**-**
*Massilia sp*. OS258	ND	ND	ND	ND	**-**	**-**
*Arthrobacterscleromae*OS260	3.5 ± 0.04	1.6 ± 0.08	ND	2.1 ± 0.8	**-**	**-**
*Pseudomonas frederiksbergensis* OS261	16.9 ± 1.15	13.7 ± 1.37	0.71 ± 0.16	0.6 ± 0.2	**+**	**+**
*Flavobacterium sp*. OS262	ND	ND	7.35 ± 0.44	ND	**-**	**-**
*Flavobacterium sp*.OS263	ND	ND	ND	ND	**-**	**-**

ND–not detected; trp- tryptophan 500 μg/m

### Germination assay and plant experiments

Seed germination experiments at 15°C demonstrated the detrimental effect of chilling on germination where 50% of the control seeds failed to germinate. Results of the germination experiment were compared with early seedling growth under chilling. From the results of both experiments, strains that showed consistent increase in germination and plant growth at 15°C were *Pseudomonas frederiksbergensis* OS211, *Flavobacterium glaciei* OB146, *Pseudomonas vancouverensis* OB155, and *P*. *frederiksbergensis* OS261 (S2–S4 Figs). These strains were chosen for studying their effect in tomato grown under greenhouse conditions.

**Fig 3:**
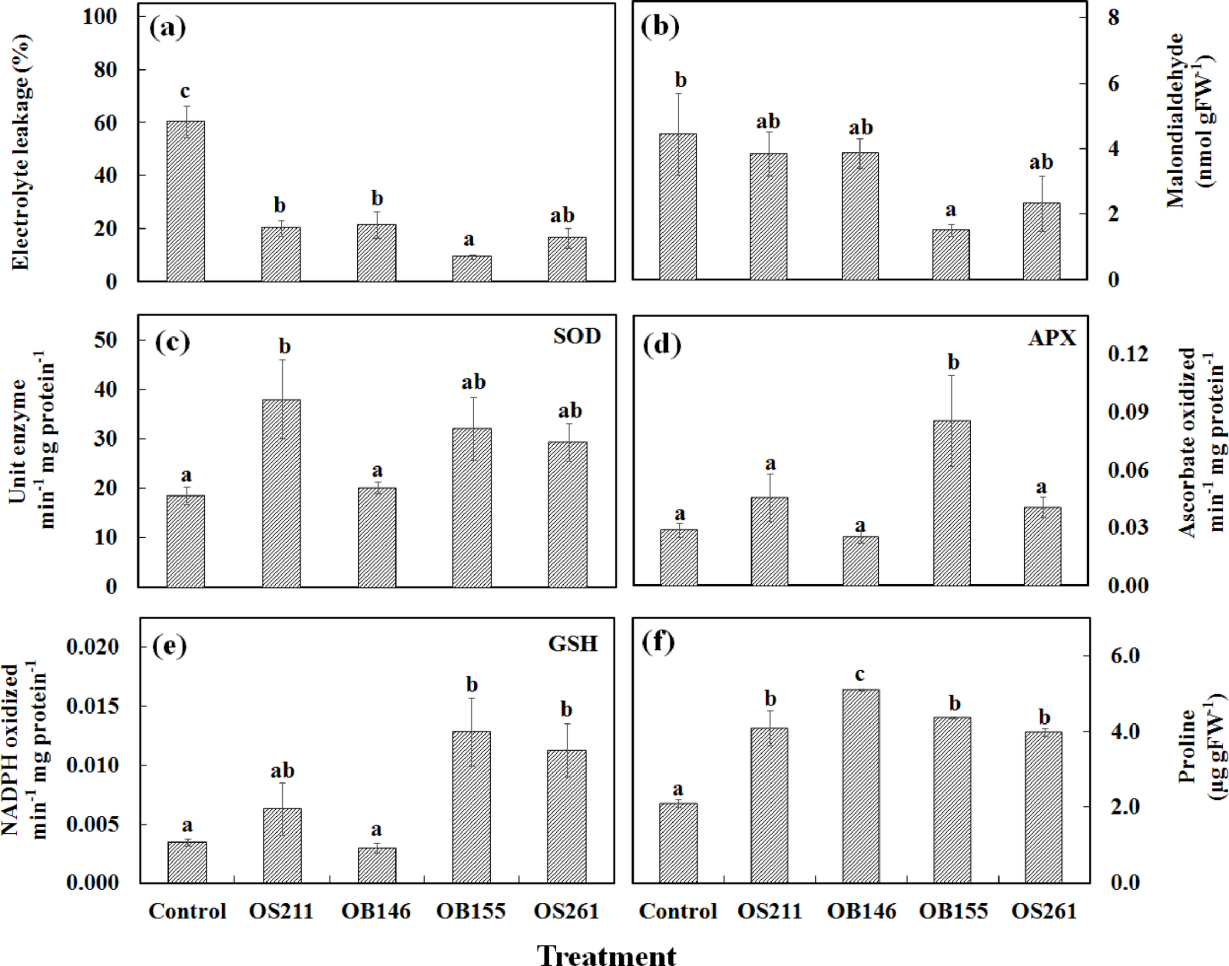
(a) Malondialdehyde content; (b) electrolyte leakage in leaves after one week of chilling treatment. Antioxidant enzyme activity in leaf samples at the end of chilling treatment (c) superoxide dismutase; (d) ascorbate peroxidase; (e) glutathione reductase; (f) Proline content after one week of chilling treatment. Alphabets above the columns indicate statistical grouping based on t-Test (LSD) with *p*≤0.05.

At the end of one week chilling treatment at 15/ 15°C, plant parameters that illustrate the extent of chilling damage and antioxidative components of the plants were studied. Electrolyte leakage and malondialdehyde content, which are biomarkers of chilling stress, showed a significant decline in tomato leaves of psychrotolerant bacterial treatments (Fig 3A and 3B). Among the treatments, strain *P*. *vancouverensis* OB155 significantly reduced electrolyte leakage and lipid peroxidation in leaf tissues under cold stress (Fig 3A and 3B). Moreover, *P*. *vancouverensis* OB155 and *P*. *frederiksbergensis* OS261 inoculation activated the antioxidant capacity of plants evidenced through significant increase in proline content in the leaves and induction of antioxidant enzymes SOD, APX and GSH (Fig 3C, 3D, 3E and 3F). Bacterial treatment in general, was found to decrease chilling damage and improve the antioxidant status of plants through increasing proline content and expression of antioxidant enzymes. APX and glutathione reductase expression was significantly higher in plants treated with *P*. *vancouverensis* OB155 than control plants.

## Discussion

Bacteria that are adapted to live under sub-optimal temperature conditions have been isolated from permanent and temporarily cold environments [32]. Despite abundance of publications reporting on applications of such psychrotolerant bacteria, there has been no defined method to selectively isolate psychrotolerant bacteria or identify this fraction of bacteria from the rest of soil bacterial population [12,13,15]. Moreover, isolation using culture-based techniques which make use of non-selective media such as nutrient agar, strongly limit the number of psychrotolerant or psychrophilic isolates [33–35].

Initial enrichment of the sample has been found to yield higher colony count of psychrotolerant bacteria compared to direct plating of samples [35]. Also, use of diluted complex media has been observed to yield higher number of isolates compared to the use of full strength media for isolation [20,33,35]. Minimal media supplemented with amino acids encouraged growth of Antarctic bacterium *Pseudomonas syringae* Lz4W, and amendment of defined media with yeast extract, peptone or casamino acids significantly improved the growth rate of facultative psychrophilic bacteria *Cobetia marina* L-2 when the temperature was lowered from 11 to 5°C [11,36]. Therefore, in the present study a minimal media supplemented with filter sterilized peptone as a source of amino acids was used to isolate psychrotolerant bacteria [19].

At present, availability of validated protocols to segregate psychrophiles and psychrotolerant bacteria from mesophiles or thermophiles is strictly limited. Conclusive characterization as psychrophiles, psychrotolerants or mesophiles is impractical as hitherto available data is limited to specific taxonomic groups of bacteria or their proteins. Global cellular responses expressed specifically by psychrotolerant or psychrophilic bacteria to cold stress, have not yet been reported [37]. Attempts on comparing the proteins from psychrophilic, and mesophilic bacteria have produced ambiguous results, and crystallographic structures of psychrophilic proteins indicate a high percentage similarity in their conformation to their mesophilic counterparts [38]. Genetic screening of bacteria using PCR for specific marker sequences which act as signature sequences for psychrotolerants have also been carried out in the past [39,40]. However, the primers used in these PCR assays were genus or species specific, and thus cannot broadly delineate the physiological trait (psychrophile or psychrotolerants) in bacteria belonging to other genera.

Psychrotolerant bacteria possess several mechanisms that help to overcome cold stress, among which maintenance of membrane fluidity plays a major role [32,41,42]. From several reports on membrane fatty acids of cold-tolerant bacteria and our preliminary investigations, it was observed that, fatty acids of cold adapted bacteria have lower chain length, predominance of unsaturated C_16_ and C_18_ fatty acids and reduction in saturated long chain fatty acids. In the present study, we also observed these characteristics in the membranes of our isolates (Fig 2). Abundance of unsaturated fatty acids demonstrate the constitutive expression of fatty acid desaturases in the bacterial cells, which is a characteristic of psychrotolerant bacteria. Maximum growth temperature of the isolates used in the study was limited to a highest of 35°C and twenty percent of the total number of isolates was not able to grow at temperatures above 30°C. Several psychrophilic and psychrotolerant bacteria in the past have been found to grow at temperatures as high as 35–40°C [11,43–46].

In the present study, comparative colony appearance of the same isolate at a psychrotolerant (5°C) as well as mesophilic temperatures (25°C) was analyzed. Though psychrotolerant or psychrophilic bacteria compared to mesophiles are well adapted to low temperatures and are expected to grow proficiently under cold, the difference in time taken for growth by a psychrotolerant may not be dramatically less than a mesophile at low-non freezing temperatures [47]. Results from the present study also show such trends. However, our isolates grew relatively faster with most of the isolates showing appearance of visible colonies at 2 to 3 days of incubation at 5°C. Strains such as *Flavobacterium* sp. OR201, OR306, OB148 and *Arthrobacter scleromae* OR302, OB149 which required longer time of growth at 25°C took much similarly long periods for growth at 5°C (Fig 2). The above mentioned growth physiology, combined with the synthesis of short chain fatty acids and constitutive expression of unsaturated fatty acids indicate the psychrotolerant nature of isolates obtained in the present study.

Majority of the isolates from Arctic and Antarctic permafrost are psychrotolerant in nature rather than psychrophiles [48]. The diversity of the cultured bacteria in the present work are covered across genera *Pseudomonas*, *Flavobacterium*, *Arthrobacter*, *Massilia*, *Pedobacter*and *Flavimonas*. Representatives of Gammaproteobacteria, Actinobacteria, Firmicutes, and Bacteroidetes to which the isolated strains represent, have been observed to colonize cold environments [32,34]. Several representatives of genus *Arthrobacter* and *Pseudomonas* have been isolated from Arctic and Antarctic environments [16,23].

Malate and oxalate have been reported to be major constituents of plant root exudates and bacteria that are able to colonize root surfaces are found to utilize these compounds as sources of carbon [49]. Among our isolates strains belonging to genus *Arthrobacter*, *Flavimonas*, *Flavobacterium*, *Massilia* and *Pseudomonas* were able to utilize malate. Strains of genus *Massilia* and *Pseudomonas* were positive for oxalate utilization (S1 Fig). Therefore, these bacteria can be screened for potential use as plant colonizing beneficial bacteria to improve plant growth or protect against pathogen attack. In plant experiments, strains *Pseudomonas frederiksbergensis* OS211, *Flavobacterium glaciei* OB146, *Pseudomonas vancouverensis* OB155, and *P*. *frederiksbergensis* OS261 were used as inoculants for seeds of tomato.

Reactive oxygen species (ROS) are highly reactive and detrimental metabolic products which have well been described to be associated with temperature induced stress in plants [5, 7]. Moreover, they also control expression of several genes controlling processes such as growth, hypersensitive response leading to programmed cell death (PCD), defense as well as stress responses and development. Recently, a maize mitogen-activated protein kinase kinase (MAPKK) gene *ZmMKK1* was reported to induce chilling tolerance when expressed in tobacco by improving antioxidant enzyme activity, accumulation of osmolytes and significant increase in expression of ROS-related genes [50]. Other strategies used by plants such as accumulation of melatonin and sugars raffinose also confer chilling tolerance through the ROS scavenging mechanism [51,52]. Therefore chilling tolerance can at least be partially attributed to improvement of ROS scavenging activity in the plant. Several studies have demonstrated the effect of psychrotolerant bacteria in alleviating chilling stress in plants [14,17,30,53,54,55]. Though not reported directly, from the results of these studies it can be observed that plant growth promoting bacteria in most cases have often been involved in the activation of ROS scavenging molecules such as proline, antioxidant enzymes and reduction of plant cell membrane damage. *Burkholderia phytofirmans* PsJN significantly reduced H_2_O_2_ content in grapevine leaves and also reduced aldehydes and malondialdehyde content which are biomarkers of ROS stress [54]. Other physiological changes reported are increase in total phenolic compounds, modification of sugar metabolism to accumulate certain sugars, maintaining of photosynthetic capacity and activation of CBF genes (C-repeat binding factor) [14,17,53].

The present study consolidates the culturable diversity of psychrotolerant bacteria from soil and their cellular and physiological adaptations to low temperatures. Inoculation of seeds of tomato with plant growth promoting psychrotolerant bacteria significantly improved plant height, root length, membrane damage in leaf tissues as evidenced through electrolyte leakage and malondialdehyde content. Further, the antioxidant enzyme activity was improved in plants; the factors combined can be taken as steps towards ROS scavenging in the plants.

## Supporting Information

S1 Fig Germination percentage of tomato seeds subjected to chilling stress at 15°C. (TIF)

S2 Fig Shoot length tomato seedlings subjected to one week of chilling stress after treatment with psychrotolerant bacterial isolates. Treatment columns with (*) marked isolates were taken for pot experiments.
(TIF)

S3 Fig Root length tomato seedlings subjected to one week of chilling stress after treatment with psychrotolerant bacterial isolates. Treatment columns with (*) marked isolates were taken for pot experiments.
(TIF)

S4 Fig Carbon source utilization pattern of the psychrotolerant bacterial isolates at genus level observed through utilization of sugars, sugar alcohols, organic acids. (TIF)

S1 Table Basic biochemical characteristics of the isolates observed at 5°C. (DOCX)
