# Supplementary figures and images for: Cold Stress Tolerance in Psychrotolerant Soil Bacteria and Their Conferred Chilling Resistance in Tomato (Solanum lycopersicum Mill.) under Low Temperatures
Source: PLoS One. 2016 Aug 31;11(8):e0161592. doi: 10.1371/journal.pone.0161592 (PMC5006972; doi:10.1371/journal.pone.0161592)

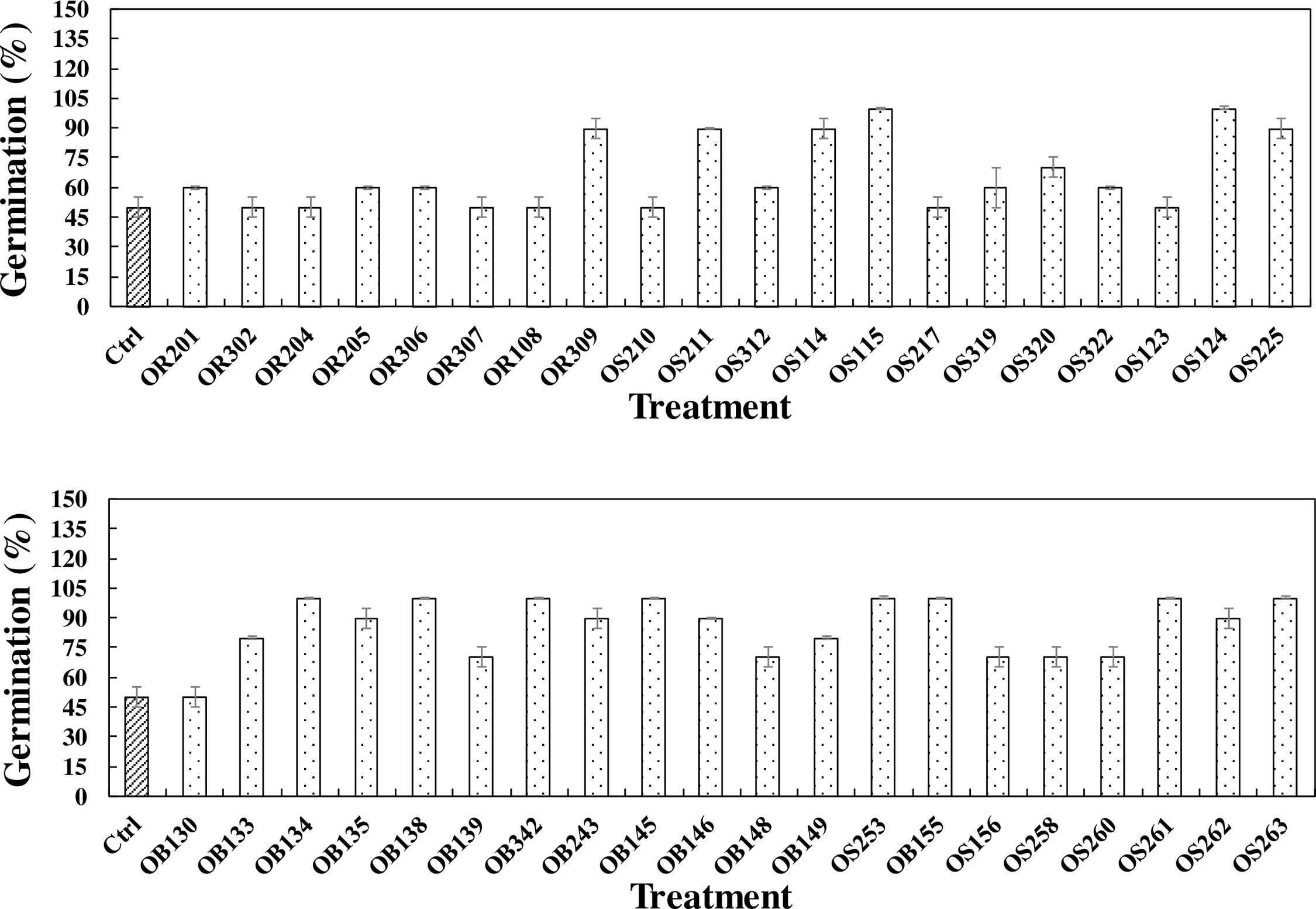

Supplement: S1 Fig — (TIF) [file pone.0161592.s001.tif]

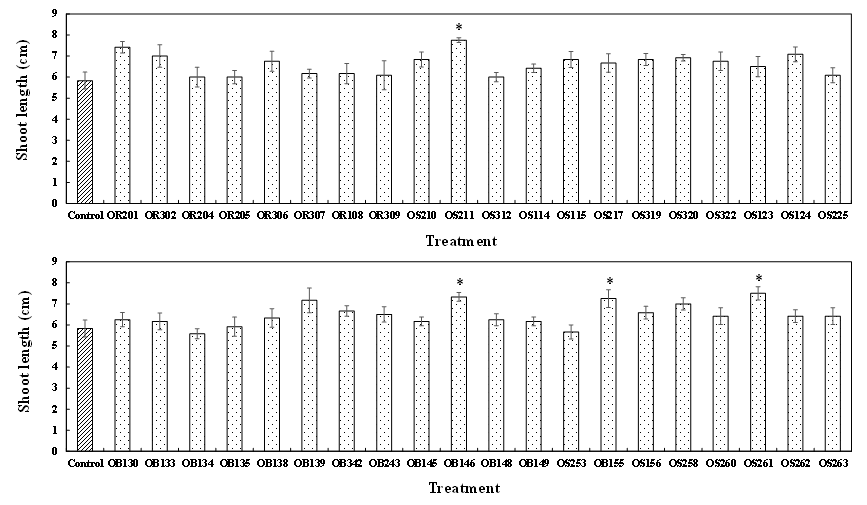

Supplement: S2 Fig — Treatment columns with (*) marked isolates were taken for pot experiments. (TIF) [file pone.0161592.s002.tif]

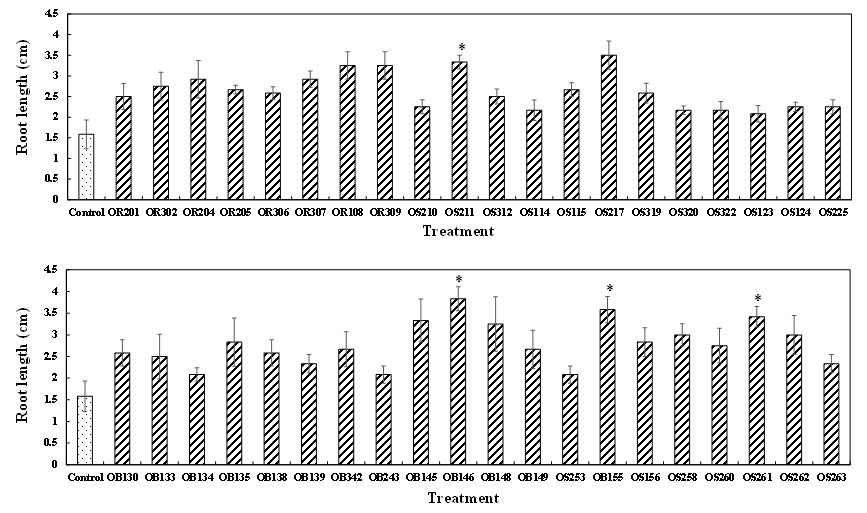

Supplement: S3 Fig — Treatment columns with (*) marked isolates were taken for pot experiments. (TIF) [file pone.0161592.s003.tif]

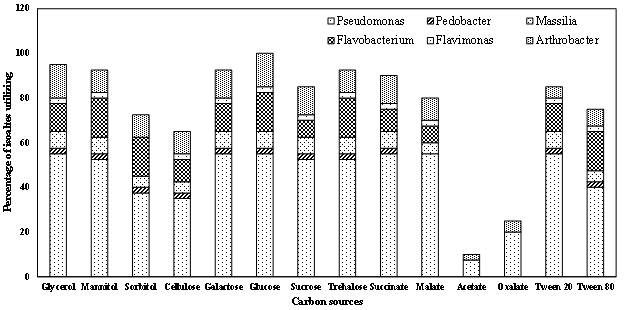

Supplement: S4 Fig — (TIF) [file pone.0161592.s004.tif]
