# Supplementary material for: Cold Stress Tolerance in Psychrotolerant Soil Bacteria and Their Conferred Chilling Resistance in Tomato (Solanum lycopersicum Mill.) under Low Temperatures
Source: PLoS One. 2016 Aug 31;11(8):e0161592. doi: 10.1371/journal.pone.0161592 (PMC5006972; doi:10.1371/journal.pone.0161592)
Supplement: S1 Table — (DOCX) [file pone.0161592.s005.docx]

**S1 Table. Basic biochemical characteristics of the isolates observed at 5 ˚C.**

| Strain | Gram reaction | Catalase | Oxidase | Urease | Nitrate reductase |
| --- | --- | --- | --- | --- | --- |
|  |  |  |  |  |  |
| OR201 | Gm(-)ve rods | + (ve) | + (ve) | - (ve) | + (ve) |
| OR302 | Gm(+)ve rods | + (ve) | - (ve) | - (ve) | - (ve) |
| OR204 | Gm(-)ve rods | + (ve) | + (ve) | - (ve) | - (ve) |
| OR205 | Gm(+)ve rods | + (ve) | - (ve) | - (ve) | - (ve) |
| OR306 | Gm(-)ve rods | + (ve) | + (ve) | - (ve) | - (ve) |
| OR307 | Gm(-)ve rods | + (ve) | + (ve) | - (ve) | - (ve) |
| OR108 | Gm(-)ve rods | + (ve) | + (ve) | - (ve) | + (ve) |
| OR309 | Gm(-)ve rods | + (ve) | + (ve) | - (ve) | - (ve) |
| OS210 | Gm(-)ve rods | + (ve) | - (ve) | - (ve) | - (ve) |
| OS211 | Gm(-)ve rods | + (ve) | + (ve) | - (ve) | - (ve) |
| OS312 | Gm(-)ve rods | + (ve) | - (ve) | - (ve) | - (ve) |
| OS114 | Gm(-)ve rods | + (ve) | + (ve) | - (ve) | + (ve) |
| OS115 | Gm(+)ve rods | + (ve) | - (ve) | - (ve) | - (ve) |
| OS217 | Gm(-)ve rods | + (ve) | - (ve) | - (ve) | - (ve) |
| OS319 | Gm(-)ve rods | + (ve) | + (ve) | - (ve) | - (ve) |
| OS320 | Gm(-)ve rods | + (ve) | + (ve) | - (ve) | - (ve) |
| OS322 | Gm(-)ve rods | + (ve) | + (ve) | - (ve) | + (ve) |
| OS123 | Gm(-)ve rods | + (ve) | + (ve) | - (ve) | + (ve) |
| OS124 | Gm(-)ve rods | + (ve) | + (ve) | - (ve) | - (ve) |
| OS225 | Gm(-)ve rods | + (ve) | + (ve) | - (ve) | - (ve) |
| OB130 | Gm(+)ve rods | + (ve) | - (ve) | + (ve) | + (ve) |
| OB133 | Gm(-)ve rods | + (ve) | + (ve) | - (ve) | + (ve) |
| OB134 | Gm(-)ve rods | + (ve) | + (ve) | - (ve) | + (ve) |
| OB135 | Gm(-)ve rods | + (ve) | - (ve) | - (ve) | - (ve) |
| OB138 | Gm(-)ve rods | - (ve) | + (ve) | - (ve) | - (ve) |
| OB139 | Gm(-)ve rods | + (ve) | + (ve) | - (ve) | - (ve) |
| OB342 | Gm(-)ve rods | + (ve) | + (ve) | - (ve) | - (ve) |
| OB243 | Gm(-)ve rods | + (ve) | + (ve) | - (ve) | - (ve) |
| OB145 | Gm(-)ve rods | + (ve) | - (ve) | - (ve) | - (ve) |
| OB146 | Gm(-)ve rods | - (ve) | + (ve) | - (ve) | + (ve) |
| OB148 | Gm(-)ve rods | - (ve) | + (ve) | - (ve) | + (ve) |
| OB149 | Gm(+)ve rods | + (ve) | - (ve) | - (ve) | - (ve) |
| OS253 | Gm(-)ve rods | + (ve) | + (ve) | - (ve) | - (ve) |
| OB155 | Gm(-)ve rods | + (ve) | + (ve) | - (ve) | + (ve) |
| OS156 | Gm(-)ve rods | + (ve) | + (ve) | - (ve) | + (ve) |
| OS258 | Gm(-)ve rods | + (ve) | + (ve) | - (ve) | + (ve) |
| OS260 | Gm(+)ve rods | + (ve) | - (ve) | - (ve) | + (ve) |
| OS261 | Gm(-)ve rods | + (ve) | + (ve) | - (ve) | - (ve) |
| OS262 | Gm(-)ve rods | + (ve) | + (ve) | - (ve) | + (ve) |
| OS263 | Gm(-)ve rods | + (ve) | - (ve) | - (ve) | + (ve) |

+ (ve) growth; – (ve) no growth
